# Clinical efficacy and safety of two highly purified human menopausal gonadotropins in women undergoing *in vitro* fertilization

**DOI:** 10.1530/RAF-24-0132

**Published:** 2025-06-10

**Authors:** Kamini A Rao, Geeta Khanna, Himanshu Bavishi, N Sanjeeva Reddy, Kajal Mangukiya, Renu Jain, Kishan PV

**Affiliations:** ^1^ Milann Fertility, (A Unit of BACC Healthcare), Bangalore, Karnataka, India; ^2^ Ajanta Hospital & IVF Centre, Lucknow, Uttar Pradesh, India; ^3^ Bavishi Fertility Institute, Ahmedabad, Gujarat, India; ^4^ Department of Reproductive Medicine & Surgery, Sri Ramachandra Medical Centre, Chennai, Tamil Nadu, India; ^5^ Orkid Medilife Pvt Ltd, Surat, Gujarat, India; ^6^ GP Shekhawati Hospital, Jaipur, Rajasthan, India; ^7^ Sanzyme P Ltd, Hyderabad, Telangana, India

**Keywords:** controlled ovarian stimulation, Gynogen HP, human menopausal gonadotropins, in vitro fertilization, Menopur

## Abstract

**Abstract:**

This study compared the efficacy and safety of two highly purified (HP) human menopausal gonadotropin (hMG) preparations, Gynogen HP and Menopur, in women undergoing controlled ovarian stimulation (COS) for *in vitro* fertilization (IVF). A multicenter, randomized, active-controlled noninferiority comparative study was conducted between 2019 and 2021. Women aged 21–40 undergoing COS for their first or second IVF cycle, with or without intracytoplasmic sperm injection, were randomized to receive either Gynogen HP or Menopur. The primary endpoint was to determine the total number of oocytes retrieved. Key secondary endpoints included total hMG dose, days of hMG stimulation, fertilization rate, implantation rate, clinical pregnancy rate and safety. A total of 150 patients were randomized into two groups: 77 received Gynogen HP and 73 received Menopur COS. The primary endpoint, the number of oocytes retrieved (mean ± standard deviation (SD)), was similar between the Gynogen HP (6.3 ± 3.39) and Menopur (6.7 ± 4.52) groups. The least square (LS) mean values were 5.9 for the Gynogen HP and 6.3 for Menopur, with an LS mean difference of – 0.4 (95% CI: −1.83, 1.07; *P* = 0.6067), indicating that noninferiority as the lower limit of the 95% CI was above the non-inferiority threshold of −2.0. Secondary efficacy endpoints and safety analysis showed no significant differences between groups. Gynogen HP is noninferior and therapeutically equivalent to Menopur in terms of the number of oocytes retrieval, with a comparable safety profile. These results support the use of Gynogen HP for COS in women undergoing IVF as a part of assisted reproduction techniques.

**Lay summary:**

This study compared two fertility medications (Gynogen HP and Menopur) that are used to help women recruit eggs for IVF treatment. The researchers assessed if these medications are equally potent and safe. The study involved 150 women undergoing their first or second round of IVF between 2019 and 2021. Half of the women received Gynogen HP, while another half received Menopur. The main finding was that both medications worked similarly – women on Gynogen HP recruited an average of 6.3 eggs, while those on Menopur recruited an average of 6.7 eggs. This small difference was not meaningful from a medical perspective. Other important factors, such as pregnancy rates and safety concerns, were also similar between the two medications. Researchers concluded that Gynogen HP works just as well as Menopur for IVF treatment and is equally safe to use. This means doctors can confidently prescribe either medication for women undergoing IVF.

## Introduction

Infertility is a significant global health concern, affecting millions of individuals worldwide. According to the World Health Organization (WHO), approximately 17.5% of adults worldwide, roughly one in six individuals, experience infertility during their lifetime (World Health Organization 2023). Infertility, as defined by the WHO, is a disease of the male or female reproductive system characterized by the inability to achieve a pregnancy after 12 months or more of regular unprotected sexual intercourse (World Health Organization 2023). This condition often necessitates advanced interventions, including assisted reproductive technologies (ARTs) such as *in vitro* fertilization (IVF) (World Health Organization 2024). IVF, with or without intracytoplasmic sperm injection (ICSI), is a leading ART, offering hope to couples facing infertility (Choe & Shanks 2024). The success of IVF/ICSI depends on the controlled ovarian stimulation (COS) protocols using gonadotropins to induce follicular growth and oocyte maturation (Choe & Shanks 2024).

In the context of ART, gonadotropins play a crucial role in COS for women undergoing IVF, stimulating follicular development and oocyte maturation. However, the optimal preparation of gonadotropins remains an area of investigation. Human menopausal gonadotropin (hMG) preparations, which combine follicle-stimulating hormone (FSH) and luteinizing hormone (LH) activities, are essential for follicular recruitment and growth, serving as a mainstay in COS (Deeks 2018). Highly purified hMG (HP-hMG) preparations, such as Menopur, are derived from the urine of postmenopausal women and are specifically designed to optimize the balance of FSH and LH, thereby supporting folliculogenesis and steroidogenesis (Deeks 2018). Extensive research has demonstrated the clinical efficacy and tolerability of highly purified hMG, indicating comparable outcomes to recombinant FSH (rFSH) and potential improvements in certain aspects of embryo quality within the IVF setting (Mannaerts *et al.* 1991, Deeks 2018).

The preference for HP-hMG among reproductive specialists worldwide stems from its distinct endocrine profile compared to rFSH, which may better mimic the physiological hormonal milieu and influence ART outcomes (Deeks 2018). In addition, HP-hMG has been associated with optimal follicular responses and a reduced risk of ovarian hyperstimulation syndrome (OHSS), a significant concern in COS (Deeks 2018).

This study aims to compare the clinical efficacy and tolerability of two HP-hMG preparations: Gynogen HP (Sanzyme P Ltd) and Menopur (Ferring Pharmaceuticals Inc, Germany), both administered subcutaneously (SC) in women undergoing IVF. This study seeks to provide robust comparative data that may inform clinical decisions, refine COS strategies and establish Gynogen HP as an effective alternative to Menopur for COS in women undergoing IVF.

## Materials and methods

### Study design

This multicenter, prospective, randomized, open-label, noninferiority, active-controlled, parallel-group comparative study was conducted between 2019 and 2021 at six sites across India. Clinical and laboratory procedures were standardized across the study centers to minimize variability by ensuring uniformity in protocols, training, and equipment usage. This included uniform protocols for COS, follicular monitoring, oocyte retrieval, sperm preparation, and embryo culture. Clinicians and embryologists received training to ensure consistency in oocyte grading, fertilization assessment, and embryo scoring. The laboratories of all the participating sites were accredited by the National Accreditation Board for Testing and Calibration Laboratories (NABL). The study adhered to the Declaration of Helsinki, the International Conference on Harmonization Guidelines for Good Clinical Practice, and local regulatory requirements (ICH). The study protocol received approval from the Central Drugs Standard Control Organisation (CDSCO) and respective institutional ethics committees of the participating centers. The study was prospectively registered with the Clinical Trials Registry India (CTRI/2019/10/021801). The participating centers, geographically distributed across India as per CDSCO regulations, which included: Milann Fertility (unit of BACC healthcare) in Bangalore; Ajanta Hospital and IVF Center in Lucknow; Bavishi Fertility Institute Private Limited in Ahmedabad; Sri Ramachandra Medical College and Research Institute in Chennai; Orkid Medlife Pvt. Ltd in Surat; and GP Shekhawati Hospital and Research Center in Jaipur.

### Study population

The study enrolled women aged 21–40 years, undergoing COS for their first or second IVF cycle, with or without intracytoplasmic sperm injection (ICSI). Being a phase 3 controlled clinical study, the study aimed to ensure a homogeneous cohort with favorable prognostic factors. Eligible patients were required to have a body mass index (BMI) ranging from 18.5 to 30 kg/m^2^, normal basal levels of FSH, LH, estradiol (E2), progesterone, and prolactin, and an antral follicle count of 6–10. Patients exhibited regular ovulatory cycles (21–35 days) and underwent transvaginal ultrasonography (TVUS) confirming normal uterine and adnexal anatomy. Furthermore, patients exhibited either normal or clinically insignificant values in hematological and blood chemistry parameters. Finally, inclusion required a male partner with normal sperm motility and sperm count. Exclusion criteria included ≥2 failed hMG/human chorionic gonadotropin (hCG) induction cycles, ovarian failure, poor response history, polycystic ovary syndrome (PCOS), untreated hydrosalpinx, advanced endometriosis, abnormal endometrial pathology, large submucosal fibroids, tubal disorders, prior OHSS, drug allergies, bleeding disorders, malignancy, metabolic conditions, or recent study participation. Pregnancy, lactation, substance abuse, and positive infectious disease screening also precluded enrollment. Consent was obtained from all participants after explaining the study’s purpose and procedures.

### Objectives

The primary objective was to compare the clinical efficacy of two different HP-hMG preparations (Gynogen HP and Menopur) administered subcutaneously to female patients undergoing COS for IVF, focusing on oocyte maturation and retrieval. The noninferiority design chosen as hMG preparations (Menopur, Merional-HG and Meriofert) were widely used by reproductive specialists for COS. Menopur was selected as an active comparator as it is the innovator and only approved reference drug in the European Union and United States and has shown significant clinical outcomes in previous studies (Deeks 2018).

### Study duration

**Figure 1:**
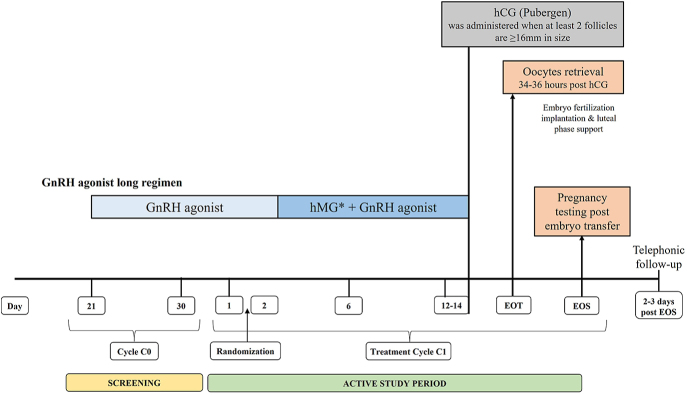
Study design. *hMG: Gynogen HP or Menopur; C0, previous menstrual cycle; C1, current menstrual cycle; EOS, end of study; EOT, end-of-treatment; GnRH, gonadotropin-releasing hormone; hCG, human chorionic gonadotropin; hMG, human menopausal gonadotropin; HP, highly purified.

The total study duration was approximately 24 months, with each patient’s participation lasting about 8–12 weeks. This included a screening period of up to 1 week, a treatment period of 4–6 weeks, and 2–4 weeks for an end-of-study (EOS) visit following treatment. Patient participation commenced on day 21 of their previous menstrual cycle (cycle 0). A detailed study design schematic is presented in Fig. 1.

### Study treatments

Open-label randomization (1:1) was performed after all the screening-related activities were completed on day 21 of the previous menstrual cycle (cycle 0). As per the randomization, participants received either Gynogen HP or Menopur using the interactive voice response system (IVRS). A patient was considered randomized when a patient had met all the eligibility criteria and received a randomization number from IVRS. The randomization was stratified by age (≤35 years and >35 years) and BMI (18.5 – <25.0 kg/m^2^ and ≥25.0 kg/m^2^). The open-label study design was chosen as a pragmatic choice due to logistical and operational constraints, such as differences in packaging, formulation and administration protocols between Gynogen HP and Menopur. Clinicians were aware of the assigned medication when deciding the starting dose (150–225 international units (IUs)), but dosing was guided by a standardized protocol based on patient age, BMI, and ovarian reserve (antral follicle count and basal FSH). Dose adjustments were made objectively based on ovarian response (E2 levels and TVUS findings), reducing the risk of bias. In addition, to ensure unbiased analysis, clinical data analysts were kept blinded to treatment allocation throughout the study by concealing the randomization code. All study outcome parameters and endpoints were ‘indisputably objective’ in nature rather than subjective, ensuring that no bias affected the endpoints considered in this open-label clinical study.

Enrolled patients underwent downregulation using a standard gonadotropin-releasing hormone (GnRH) agonist long protocol with leuprolide 0.5 mg starting from day 21 of the previous menstrual cycle C0. After the downregulation, a starting dose of either Gynogen HP or Menopur in the range of 150–225 IUs was maintained for approximately first 4–5 days of the current menstrual (cycle 1) starting from day 1 or 2. After this period, dose adjustments were allowed depending on the ovarian response, monitored by serum E2 levels and ultrasonographic measurement. The dose of leuprolide acetate was decreased to 0.25 mg starting on day 3 of cycle 1 and continued daily until the day of hCG administration. The hMG plus GnRH agonist was continued until at least two follicles ≥16 mm in diameter were observed, or as per the investigator’s discretion. A single trigger of hCG Pubergen (5,000–10,000 IU), or as per the investigator’s clinical judgment, was given approximately on cycle 1 day 12–14, once at least two follicles achieved a size of ≥16 mm, as assessed by TVUS.

The long protocol was selected based on its use in a prior study comparing Menopur with Merional (Alviggi *et al.* 2013), ensuring alignment with established protocols for the reference product (Menopur) and supporting regulatory and export purposes. This protocol was chosen for its established efficacy in achieving pituitary downregulation, minimizing premature LH surge, and optimizing oocyte yield in good prognosis patients, aligning with standard practices in India during the study period (2019–2021).

### Outcome measures

The primary efficacy outcome of the study was the total number of oocytes retrieved in 34–36 h after hCG administration, performed via ultrasound follicular aspiration.

Secondary efficacy assessments included evaluation of the total hMG dose, duration of hMG stimulation, serum E2 concentrations, cancellation of IVF cycle, number of mature follicles (≥16 mm in diameter on the day of hCG administration), number of mature oocytes, number of inseminated oocytes, fertilization rates, number of viable embryos, embryo score, number of transferred embryos, implantation rates, and clinical pregnancy rates.

Safety assessments were performed during the entire study at each visit. Adverse events (AEs) and serious AEs (SAEs) experienced by patients were recorded and reported. SAE information was collected at every study visit from screening through the end of the study. In addition to data collected for AEs, hospital discharge summaries and external laboratory reports were reviewed, wherever possible, to provide supporting information for each SAE.

### Statistical analysis

Per-protocol (PP) set was used for all efficacy analyses, and the safety analysis set was used for safety and tolerability analyses. The safety analysis set included all patients who received at least one dose of the study drug. Safety data were presented according to the actual treatment received. All analyses were measured using the SAS^®^ version 9.4. Sample size was calculated based on a noninferiority design for the primary endpoint (the number of oocytes retrieved) with *α* = 0.05, *β* = 0.20 and a coefficient of variation of 0.40 based on a previously observed mean of ten oocytes retrieved with a SD of 4; 144 patients (72 per treatment group) randomized in a 1:1 ratio would provide 80% power for the primary endpoint analysis using one-way analysis of variance (ANOVA).

The primary outcome was analyzed using ANOVA with treatment, age stratification (≤35 years, >35 years), and BMI stratification (18.5 – <25.0 kg/m^2^, ≥25 kg/m^2^) as factors. The estimated least square (LS) means for both treatment groups and their difference were reported with a two-sided 95% confidence interval (CI). Gynogen HP was considered noninferior to Menopur if the lower limit of the 95% CI was greater than −2.0. All efficacy parameters were tested using two-sided tests with a 5% significance level, unless specified otherwise. Continuous data were summarized using descriptive statistics (*n*, mean, SD, median, minimum, and maximum). Discrete data were summarized using frequency counts and percentages.

## Results

### Patient disposition

**Figure 2:**
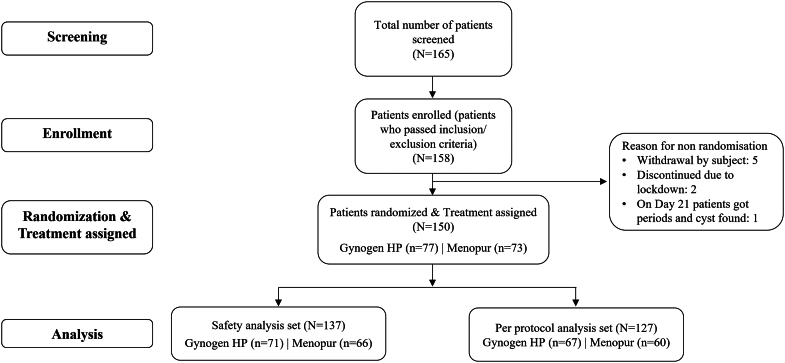
Patient disposition.

Of 158 patients enrolled in the study, 150 were randomized to receive Gynogen HP (*n* = 77) or Menopur (*n* = 73), which is the intent-to-treat population set that includes all patients randomized and assigned to hMG treatment. The safety analysis set includes all patients who were randomized and received at least one dose of the study drug – hMG comprised of 137 (Gynogen HP, *n* = 71 and Menopur, *n* = 66) evaluable patients. The PP analysis set includes all patients in the safety analysis set except those excluded as a result of a major protocol violations that impact the efficacy analysis and comprised of 127 (Gynogen HP, *n* = 67 and Menopur, *n* = 60) evaluable patients. A total of ten subjects had major protocol deviations (four in Gynogen and ix in Menopur treatment groups). The reasons for major protocol deviations included history of hypothyroidism during screening, EOS/end-of-treatment (EOT) visits missed, principal investigator froze the embryos, and tests performed in local laboratories due to COVID-19 crisis. The flowchart for patient disposition is shown in Fig. 2.

### Baseline demographics and clinical characteristics

The summary of study patients’ demographics and baseline clinical characteristics are presented in Table 1. The mean age for patients in both treatment groups was around 30 years, exhibiting a comparably distributed age range. Specifically, patients in the Gynogen HP group demonstrated a mean age of 30.3 years (SD: 4.22), spanning from 21 to 40 years, while those in the Menopur group mirrored this average age at 30.3 years (SD: 4.30), with an age range of 21–38 years. Likewise, the BMI measurements were also similar between the two groups. Patients in the Gynogen HP group presented with a mean BMI of 25.0 kg/m^2^ (SD: 3.13), ranging from 18.7 to 29.9 kg/m^2^, whereas their counterparts in the Menopur group displayed a mean BMI of 24.7 kg/m^2^ (SD: 2.81), with a BMI range of 18.9–30.1 kg/m^2^.

**Table 1:** Baseline demographics and clinical characteristics (safety analysis set).

Characteristics	Gynogen HP	Menopur
*n*	71	66
Age (years)		
Mean ± SD	30.3 ± 4.22	30.3 ± 4.30
Min, max	21, 40	21, 38
BMI (kg/m^2^)		
Mean ± SD	25.0 ± 3.13	24.7 ± 2.81
Min, max	18.7, 29.9	18.9, 30.1
Patients with previous pregnancies, *n* (%)	1 (1.4)	9 (13.6)
Normal deliveries	0	4 (6.1)
Spontaneous miscarriages	1 (1.4)	4 (6.1)
Other events	0	1 (1.5)

BMI, body mass index; HP, highly purified; SD, standard deviation.

However, there were differences between the groups in terms of reproductive history. Only one patient (1.4%) within the Gynogen HP group reported a previous pregnancy, compared to nine patients (13.6%) in the Menopur group who had a history of prior pregnancies. Furthermore, the Menopur group exceeded the Gynogen HP group in both normal deliveries (6.1 versus 0.0%) and occurrences of spontaneous miscarriages (6.1 versus 1.4%).

### Primary endpoint: total number of oocytes retrieved

**Figure 3:**
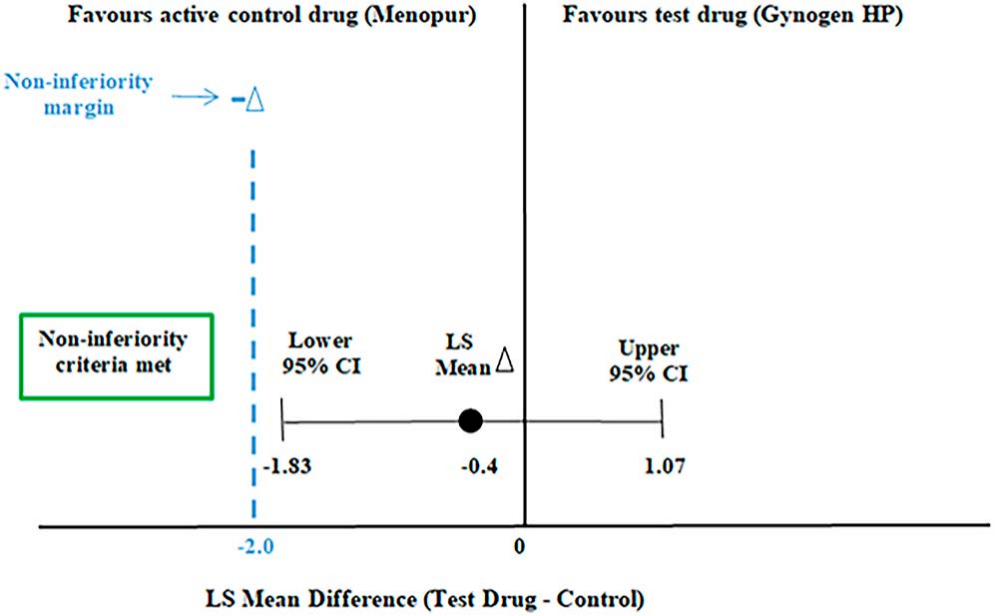
Primary endpoint – noninferiority between Menopur and Gynogen HP. CI, confidence interval; HP, highly purified; LS, least squares.

The PP set for the primary endpoint analysis included 118 evaluable patients out of 127 total PP patients: 60 in the Gynogen HP group and 58 in the Menopur group. The mean number of oocytes retrieved (SD) was 6.3 (3.39) in the Gynogen HP group and 6.7 (4.52) in the Menopur group, as shown in Table 2. The LS mean values were calculated at 5.9 and 6.3 in the Gynogen HP and Menopur groups, respectively, with a LS mean difference of −0.4 observed in the number of oocytes retrieved. The 95% CI for this difference ranged from −1.83 to 1.07. With a *P*-value of 0.6067, the difference between the two groups was not statistically significant, indicating the groups were comparable (Table 2 and Fig. 3). In the PP set, since the lower limit of the 95% CI of the LS mean difference was greater than the noninferiority limit, i.e. −2.0, Gynogen HP can be claimed noninferior to Menopur in terms of efficacy.

**Table 2:** Primary endpoint: the number of oocytes retrieved (per protocol set).

Parameter	GHP (*n* = 67)	MP (*n* = 60)	GHP vs MP, LSM*	95% CI	PNI limit	*P*
Total number of oocytes retrieved			−0.4	−1.83, 1.07	−2.0	0.6067
*n*	60	58				
Mean ± SD	6.3 ± 3.39	6.7 ± 4.52				
LSM ± SE*	5.9 ± 0.67	6.3 ± 0.66				

* The LSM of the number of oocytes retrieved and the difference between the treatments are estimated using ANOVA, with treatment and age groups (≤35 years, >35 years) used as factors.

LSM, least square mean; CI, confidence interval; HP, highly purified; SD, standard deviation; SE, standard error; GHP, Gynogen HP; MP, Menopur; PNI, predefined noninferiority; ANOVA, analysis of variance.

Subgroup analysis by age (≤35 years, >35 years) and BMI (18.5 – <25.0 kg/m^2^ and ≥25.0 kg/m^2^) are summarized in Table 3. For patients aged ≤35 years, the mean number of oocytes retrieved was 6.3 (SD: 3.45) for Gynogen HP and 7.0 (SD: 4.62) for Menopur (mean difference: −0.6; 95% CI: −2.22, 0.98; *P* = 0.4443). For patients aged >35 years, the mean was 6.3 (SD: 3.15) for Gynogen HP and 5.0 (SD: 3.55) for Menopur (mean difference: 1.3; 95% CI: −2.48, 5.05; *P* = 0.4738). For BMI ≥25.0 kg/m^2^, the mean was 6.5 (SD: 3.85) for Gynogen HP and 7.6 (SD: 5.07) for Menopur (mean difference: −1.1; 95% CI: −3.46, 1.28; *P* = 0.3590). No statistically significant differences were observed in any subgroup, consistent with the study’s power for the overall population rather than subgroups.

**Table 3:** Subgroup analyses of primary endpoint, the number of oocytes retrieved (per protocol set), by age and BMI.

Subgroup	Gynogen HP (*n* = 67)	Menopur (*n* = 60)	*P*	MD (95% CI)
Age: ≤35 years			0.4443	−0.6 (−2.22, 0.98)
*n*	53	50		
Mean (SD)	6.3 (3.45)	7.0 (4.62)		
Age: >35 years			0.4738	1.3 (−2.48, 5.05)
*n*	7	8		
Mean (SD)	6.3 (3.15)	5.0 (3.55)		
BMI: 18.5–<25.0 kg/m^2^			0.8203	0.2 (−1.58, 1.99)
*n*	29	32		
Mean (SD)	6.2 (2.88)	6.0 (3.95)		
BMI: ≥25.0 kg/m^2^			0.3590	−1.1 (−3.46, 1.28)
*n*	31	26		
Mean (SD)	6.5 (3.85)	7.6 (5.07)		

BMI, body mass index; CI, confidence interval; HP, highly purified; SD, standard deviation; MD, mean difference.

### Secondary efficacy endpoints

The secondary efficacy analysis evaluated continuous variables such as the total hMG dose, number of days of hMG stimulation, serum E2 concentration, and embryo score. The mean values for Gynogen HP versus Menopur were as follows: total hMG dose (2,712.3 IU versus 2,685.0 IU), number of days of hMG stimulation (11.4 versus 11.1 days), and serum E2 concentration (1,838.06 pg/mL versus 2,234.02 pg/mL, respectively). These parameters were closely aligned between the two groups, with no statistically significant differences observed, as indicated by *P*-values >0.05 (Table 4).

**Table 4:** Analysis of secondary efficacy endpoints (per protocol set). Data are presented the mean ± SD or as *n* (%).

Parameter	Gynogen HP (*n* = 67)	Menopur (*n* = 60)	*P*
Total hMG dose (IU)			0.8503*
*n*	67	60	
Mean ± SD	2,712.3 ± 890.15	2,685.0 ± 715.76	
Number of days of hMG stimulation			0.3924*
*n*	60	58	
Mean ± SD	11.4 ± 1.67	11.1 ± 1.87	
Serum E2 concentration (pg/mL)			0.1839*
*n*	42	43	
Mean ± SD	1,838.06 ± 1,150.60	2,234.02 ± 1,540.94	
Embryo score			0.9650*
*n*	56	48	
Mean ± SD	1.7 ± 1.05	1.7 ± 1.01	
Mature follicles of diameter ≥16 mm^‡^	57 (85.1)	54 (90.0)	0.4037^†^
Mature oocytes (metaphase II)^§^	58 (86.6)	53 (88.3)	0.7646^†^
Failed to achieve mature oocytes	9 (13.4)	7 (11.7)	0.7646^†^
At least one inseminated oocyte	58 (86.6)	53 (88.3)	0.7646^†^
At least one viable embryo	56 (83.6)	48 (80.0)	0.6007^†^
At least one embryo transferred	53 (79.1)	48 (80.0)	0.9006^†^
Fertilization per inseminated oocyte	251/309 (81.2)	312/347 (89.9)	0.0015^†^
Implantation per embryo transferred	15/139 (10.8)	23/126 (18.3)	0.0834^†^
Clinical pregnancy per embryo transferred	13/139 (9.4)	19/126 (15.1)	0.1531^†^
Clinical pregnancy per oocyte retrieved	13/380 (3.4)	19/388 (4.9)	0.3061^†^
Clinical pregnancy failure per embryo transferred	126/139 (90.6)	107/126 (84.9)	0.1531^†^
IVF cancellation rate	18 (26.9)	15 (25.0)	0.8108^†^

* *P*-value was obtained using two-sample *t*-test.

^†^ *P*-value was obtained using Chi-square test or Fisher’s exact test, as appropriate. The number of days of hMG stimulation duration was calculated as (date of hCG injection – hMG treatment start date +1). Serum E2 concentration, i.e. laboratory value of serum E2 on the day or one day before or after of hCG injection, is considered for the descriptive statistics. Percentages were based on the total number of patients (*n*) in each treatment group.

^‡^ On the day or one day before of hCG administration. For six patients, hCG administered 2 or 3 days after mature follicles were observed. Such patients were not included in the summary.

^§^ All the patients with data available for mature oocytes are included in this summary. They may not be counted in the mature follicles summary if hCG administered 2 or 3 days after mature follicles were observed.

E2, estradiol; hCG, human chorionic gonadotropin; hMG, human menopausal gonadotropin; HP, highly purified; IU, international units; IVF, *in vitro* fertilization; SD, standard deviation.

The analysis of secondary efficacy endpoints for categorical variables revealed that Gynogen HP and Menopur performed comparably across most assessed parameters. The percentage of mature follicles of diameter ≥16 mm (85.1 versus 90.0%), mature oocytes (86.6 versus 88.3%), and the rates of failure to achieve mature oocytes (13.4 versus 11.7%) did not differ significantly between the two groups, with *P*-values >0.05. Similarly, the percentages of at least one inseminated oocyte (86.6 versus 88.3%), at least one viable embryo (83.6 versus 80.0%) and at least one embryo transferred (79.1 versus 80.0%) were similar for both treatment groups. A notable exception was observed in the fertilization per inseminated oocytes, where Gynogen HP had a rate of 81.2% compared to Menopur with a rate of 89.9%, with a statistically significant *P*-value of 0.0015. However, implantation rates per embryos transferred (10.8 versus 18.3%) and clinical pregnancy failure per embryos transferred (9.4 versus 15.1%), clinical pregnancy per oocytes retrieved (3.4 versus 4.9) and clinical pregnancy failure per embryos transferred (90.6 versus 84.9%) showed no significant difference. In addition, the IVF cancellation rate was comparable between the two groups (26.9 versus 25.0%; Table 4). These findings suggest that both Gynogen HP and Menopur are generally similar in efficacy for most of the secondary endpoints.

### Safety

The overall summary of AEs for the safety analysis set is presented in Table 5. Five patients (3.6%) – three (4.2%) in the Gynogen HP group and two (3.0%) in the Menopur group – experienced at least one AE. Furthermore, three patients (2.2%) – two (2.8%) in the Gynogen HP group and one (1.5%) in the Menopur group – experienced at least one treatment-emergent AE (TEAE).

**Table 5:** Overall summary of AEs. Data are presented as *n* (%). Percentages are based on the total number of patients (*n*) in each treatment group. TEAEs are defined as events with start date on or after the date of first dose of treatment or events, with start date before the date of first dose of treatment whose severity worsens on or after the date of first dose of treatment.

Parameter	Gynogen HP (*n* = 71)	Menopur (*n* = 66)
At least one AE	3 (4.2)	2 (3.0)
At least one TEAE	2 (2.8)	1 (1.5)
At least one serious TEAE	0 (0)	1 (1.5)
Treatment related TEAE	0 (0)	1 (1.5)
Grade 3 or 4 TEAEs	0 (0)	0 (0)
Treatment related serious TEAE	0 (0)	1 (1.5)
Fatal TEAEs	0 (0)	0 (0)
Treatment related fatal TEAEs	0 (0)	0 (0)
TEAEs leading to hMG dose reductions/interruptions/discontinuation	0 (0)	0 (0)
Treatment-related TEAEs leading to hMG dose reductions/interruptions/discontinuation	0 (0)	0 (0)

*n*: number of subjects with any event; grade 3: severe AE, grade 4: life-threatening or disabling AE. AE, adverse event; HP, highly purified; hMG, human menopausal gonadotropin; TEAE, treatment-emergent adverse event.

In the Menopur group, one grade 2 TEAE was reported, categorized under the system organ class (SOC) for reproductive system and breast disorders as OHSS. In the Gynogen HP group, two grade 1 TEAEs were reported: pyrexia, classified under SOC general disorders and administration site conditions, and dizziness, under SOC nervous system disorders.

No grade 3 or 4 TEAEs were reported, nor were there any fatal TEAEs in the study. In addition, there were no TEAEs that led to dose reductions, interruptions, or discontinuation of hMG. There were no deaths during the course of the study. However, one patient (1.5%) experienced a serious TEAE (OHSS) that was considered related to the study treatment (Menopur), as indicated in Table 5.

## Discussion

This noninferiority study compared the clinical efficacy and tolerability of two HP-hMG preparations, Gynogen HP and Menopur. Our findings demonstrate that Gynogen HP is noninferior to Menopur regarding the total number of oocytes retrieved and for most secondary efficacy endpoints, i.e., stimulation duration, mean embryo score, mature follicles ≥16 mm, metaphase II oocytes, viable embryo transfers, and clinical pregnancy rates. The comparable efficacy and safety of Gynogen HP and Menopur provide clinicians with flexibility in selecting hMG preparations based on patient needs and practical considerations.

The HP-hMG preparations have more hCG and less LH than traditional hMG, providing the necessary LH activity for oocyte maturation. Furthermore, it has proven to be effective and safe in COS for IVF over decades. Studies show that HP-hMG (such as Menopur (Ferring) and Merional-HG/Meriofert (IBSA)) is at least as effective as rFSH for pregnancy and live-birth rates (Al-Inany *et al.* 2009, van Wely *et al.* 2011, Bordewijk *et al.* 2019).

In our study, the mean number of oocytes retrieved was comparable between the Gynogen HP (6.3) and Menopur (6.7) groups, with no statistically significant difference. This aligns with previous studies comparing different gonadotropin preparations in IVF cycles. For instance, Levi Setti *et al.* reported similar oocyte retrieval ranges when comparing hMG (6.8–14.4) with rFSH (7.2–12.9) in a large meta-analysis (Levi Setti *et al.* 2015). Our findings further support the notion that HP-hMG preparations, such as Gynogen HP, can achieve comparable outcomes to well-established preparations such as Menopur. While there is no consensus on the ideal number of oocytes for IVF success, the oocyte retrieval results for both Gynogen HP (6.3) and Menopur (6.7) fall within the optimal range of 6–15 oocytes suggested by several studies for IVF success, indicating that both treatments can provide an adequate oocyte yield for IVF procedures (Sunkara *et al.* 2011, Alviggi *et al.* 2013, Ji *et al.* 2013, Turhan *et al.* 2013, Witz *et al.* 2020).

Subgroup analyses by age and BMI showed no statistically significant differences in oocyte retrieval between Gynogen HP and Menopur, consistent with the study’s primary endpoint. Numerical differences in subgroups (e.g., higher mean oocytes with Gynogen HP in women >35 years) were not significant and should be interpreted cautiously, as the study was not powered for subgroup analyses. Larger studies are needed to explore potential differences in specific patient populations.

Gynogen HP and Menopur demonstrated comparable efficacy (*P* >0.05) across most secondary endpoints and align with previous studies (Smitz *et al.* 2007, Devroey *et al.* 2012). These included stimulation duration (11.4 versus 11.1 days) (Devroey *et al.* 2012), mean embryo score (1.7 for both), mature follicles ≥16 mm (85.1 versus 90.0%), and mature metaphase II oocytes (86.6 versus 88.3%). Both products achieved high rates of inseminated oocytes (86.6 versus 88.3%) and viable embryo transfers (83.6 versus 80.0%). The clinical pregnancy rate per oocyte retrieved was 3.4 for Gynogen HP and 4.9 for Menopur. Notably, both products’ viable embryo rates (Gynogen HP: 83.6%; Menopur: 80.0%) significantly exceeded the 54% reported in a recent Chinese study using HP-hMG products (Ji *et al.* 2020).

The Menopur group demonstrated higher fertilization rates per inseminated oocyte versus Gynogen HP (89.9 versus 81.2%, *P* = 0.0015). This difference was not attributable to male factors as baseline semen parameters were within the normal limits in both the groups. While noteworthy, this difference did not manifest in downstream outcomes, as implantation and clinical pregnancy rates remained comparable between groups.

The safety profiles of Gynogen HP and Menopur were reassuringly similar, with low incidences of AEs in both groups. Overall, 3.6% of patients experienced at least one AE, with a slightly higher incidence in the Gynogen HP group (4.2%) compared to Menopur (3.0%). TEAEs were mild-to-moderate, with no grade 3 or 4 TEAEs reported. The single case of grade 2 OHSS in the Menopur group was the only serious TEAE observed, which is within the expected range for IVF cycles (Keye *et al.* 2005).

This low incidence of OHSS in our study (one case in the Menopur group, none in the Gynogen HP group) is notable, particularly compared to studies reporting higher rates with hMG or rFSH (e.g., 9.7% in the MEGASET-HR study with Menopur) (Witz *et al.* 2020). In addition, Toftager *et al.* reported significantly higher rates of moderate/severe OHSS in their large randomized study: 10.2%/5.1% in the rFSH group and 15.6%/8.9% in the HP-hMG group (Toftager *et al.* 2016). These findings align with previous studies on HP-hMG preparations, which have reported similar safety profiles (Barrière *et al.* 2017, Blumenfeld 2018). The low incidence of OHSS in our study, particularly the absence of cases in the Gynogen HP group, further underscores the safety of the product in COS. The use of the GnRH agonist long protocol may have contributed to this low incidence by ensuring controlled pituitary suppression, potentially reducing excessive follicular recruitment compared to antagonist protocols in good prognosis patients (Al-Inany *et al.* 2016). However, the specific impact of protocol choice on OHSS risk warrants further investigation, as antagonist protocols may offer flexibility in high responders to mitigate OHSS risk (Lambalk *et al.* 2017).

Several factors (costs and insurance coverage) may influence a physician’s preference for Gynogen HP over Menopur. Gynogen HP may offer cost advantages (pharmacoeconomic advantage), making IVF more accessible to patients in low- and middle-income settings, where treatment costs are a significant barrier (Tholeti *et al.* 2024). In addition, regional supply chain dynamics and market availability may favor Gynogen HP in areas where Menopur distribution is limited or subject to supply disruptions. The absence of OHSS in the Gynogen HP group, compared to one case in the Menopur group, may also be a consideration for clinicians prioritizing safety in good prognosis patients, although the low overall OHSS incidence limits definitive conclusions. While direct cost data were not collected in this study, these potential advantages highlight the importance of considering economical and logistical factors in clinical decision-making. Future studies comparing the cost-effectiveness and real-world availability of Gynogen HP and Menopur could further inform treatment choices.

Study strengths encompass its randomized, controlled design and standardized protocols across centers, which minimized variability in clinical and laboratory procedures. However, minor inter-center differences in equipment or embryologist experience could not be fully eliminated, potentially influencing outcomes such as fertilization rates. This limitation is common in multicenter ART studies and should be considered when interpreting results. In addition, while sample size was adequate for assessing the primary endpoint, larger studies may be needed to detect potential differences in live birth rates.

In conclusion, this study demonstrated that Gynogen HP is noninferior to Menopur in terms of the number of oocytes retrieved in women undergoing IVF. Both treatments showed similar efficacy, with no significant differences across most secondary endpoints. The safety profiles of both treatments were comparable, with low incidences of AEs and treatment-emergent AEs. Overall, these results suggest that Gynogen HP is a viable alternative to Menopur, offering clinicians an effective option for COS protocols in IVF. Furthermore, while both the treatments are equally effective, Gynogen HP may provide additional benefits, such as cost savings, supply chain reliability, and consistent availability. These advantages could help alleviate the economic burden of infertility for healthcare providers and patients alike.

## Declaration of interest

KR, GK, HB, SR, KM and RJ declare no financial or other potential conflict of interest. KPV is an employee of Sanzyme P Ltd, Hyderabad, India. All the authors declare that there is no conflict of interest that could be perceived as prejudicing the impartiality of the work reported.

## Funding

This study was sponsored by Sanzyme P Ltd, Hyderabad, India.

## Author contribution statement

KAR, GK, HB, NSR and KPV contributed to the conception and design of the study. Data were acquired by KAR, GK, HB, NSR, KM, RJ, and KPV. The data were analyzed and interpreted by KAR, GK, HB, NSR, KM, RJ, and KPV. The manuscript was drafted by KAR and KPV. The manuscript was revised by KAR, GK, HB, NSR, KM, RJ, and KPV.
